# Spatial immune architecture and immunotherapy resistance in microsatellite-stable colorectal cancer

**DOI:** 10.3389/fimmu.2026.1998498

**Published:** 2026-10-26

**Authors:** Xianwei Liu, Qiangming Liao, Yuan Cheng, Hang Zhou, Riwei Wang

**Affiliations:** Department of General Surgery, Jiujiang City Key Laboratory of Cell Therapy, Jiu Jiang No.1 People’s Hospital, Jiujiang, China

**Keywords:** colorectal cancer, immunotherapy, microsatellite stability, spatial immune architecture, tumor microenvironment

## Abstract

Microsatellite-stable (MSS) colorectal cancer (CRC), which accounts for most metastatic CRC, remains largely resistant to immune checkpoint inhibitors. Although low tumor mutational burden and limited neoantigen generation contribute to this resistance, the spatial organization of the tumor microenvironment may also influence antitumor immunity. This review examines mechanisms that establish immune-resistant architectures in MSS CRC, including insufficient T-cell priming, oncogenic signaling, suppressive myeloid niches, hypoxia, abnormal vasculature, and fibroblast-driven extracellular matrix remodeling. We discuss candidate spatial biomarkers, including compartment-specific T-cell distribution, T-cell–tumor-cell distances, multicellular immune neighborhoods, perivascular niches, and the organization and localization of tertiary lymphoid structures. We also examine how fibroblast heterogeneity, collagen architecture, hyaluronan accumulation, and vascular dysfunction can restrict T-cell access and function. Emerging studies associate selected spatial features with treatment outcomes, but the supporting evidence spans small treatment-specific cohorts, mixed CRC populations, and preclinical or non-CRC models. These findings therefore support spatial profiling as a complement to molecular and clinical biomarkers rather than an independently established basis for treatment selection. Prospective studies in defined treatment settings are needed to clarify the relationship between these spatial features and immunotherapy outcomes and to assess their potential to inform combination strategies in MSS CRC.

## 1 Introduction

Colorectal cancer (CRC) represents the third most commonly diagnosed malignancy and the second leading cause of cancer-related mortality worldwide, with over 1.9 million new cases and 900,000 deaths annually (1). The therapeutic landscape for metastatic CRC (mCRC) has changed following recognition that microsatellite instability-high (MSI-H) or mismatch repair-deficient (dMMR) tumors, accounting for approximately 5% of mCRC cases (2), can show substantial sensitivity to immune checkpoint inhibitors (ICIs) targeting programmed cell death protein 1 (PD-1) or programmed death-ligand 1 (PD-L1) (3, 4). The landmark KEYNOTE-177 trial established pembrolizumab as a first-line standard for MSI-H/dMMR mCRC, demonstrating a median progression-free survival (PFS) of 16.5 months versus 8.2 months with chemotherapy (hazard ratio [HR] 0.60) (5). An unmet therapeutic need remains for the approximately 95% of patients with mCRC whose tumors are microsatellite-stable (MSS)/mismatch repair-proficient (pMMR), for whom single-agent PD-1 blockade has demonstrated minimal clinical activity, with objective response rates (ORRs) below 5% (3).

The dichotomy between MSI-H and MSS CRC has traditionally been attributed to differences in tumor mutational burden (TMB) and neoantigen load. MSI-H tumors accumulate hundreds to thousands of coding mutations due to defective DNA mismatch repair, generating abundant neoantigens that prime robust CD8^+^ T-cell responses and create a pre-existing “hot” immune microenvironment (6). By contrast, MSS tumors typically harbor lower mutation and neoantigen burdens, reducing the probability of effective spontaneous T-cell priming (7). However, mutation burden alone does not explain the heterogeneity of immune responses. First, responses have been reported with selected ICI combinations, but clinical activity varies across regimens: REGONIVO reported responses, whereas REGOMUNE did not observe objective responses (8, 9). Second, the NICHE neoadjuvant trial demonstrated pathological responses in 4 of 15 patients with pMMR early-stage colon cancer (27%) treated with ipilimumab plus nivolumab, including three major pathological responses and one partial pathological response. Baseline CD8^+^ PD-1^+^ T-cell infiltration was associated with response in this small cohort, but this exploratory finding does not establish an independently validated predictive biomarker (10). Third, spatial transcriptomic and proteomic studies in CRC have identified discrete immune-active niches, including neighborhoods at the invasive margin and organized lymphoid aggregates. These findings suggest that bulk molecular profiling may obscure local immune heterogeneity (11, 12). Tertiary lymphoid structures (TLS) provide another setting in which spatial organization may influence local immune function (13).

These observations support integrating spatial immune features with molecular and clinical biomarkers in MSS CRC immunotherapy. The spatial immune architecture, including the topological arrangement of immune, stromal, vascular, and malignant cells within tissue compartments, can influence whether effector T cells can access tumor nests, whether myeloid suppressive cells form metabolic barriers, and whether cancer-associated fibroblasts (CAFs) enforce physical exclusion through extracellular matrix (ECM) deposition (14, 15). Spatial profiling provides information about tissue context and cellular neighborhoods; longitudinal sampling is required to evaluate treatment-related changes. This review is explicitly scoped to spatially resolved or tissue-architecture-localized candidate biomarkers. We address the following core scientific questions: (i) What biological mechanisms establish immunotherapy-resistant spatial architectures in MSS CRC? (ii) Which spatial immune phenotypes are associated with response to ICIs in defined treatment settings? (iii) How do stromal and ECM barriers spatially constrain immune infiltration?

**Supplementary Table S1** provides a glossary of abbreviations and molecular symbols, summarizes the principal drug targets or mechanisms of action, and explains the clinical trial names used in this review.

## 2 Biological basis of immunotherapy resistance in MSS CRC

The resistance of MSS CRC to ICIs stems from a convergence of immunological deficits that collectively establish “cold” or “excluded” tumor microenvironments. Understanding these mechanisms provides the mechanistic foundation for interpreting spatial immune architectures as biomarkers.

The colon’s crypt-based mucosal architecture provides an anatomical framework for interpreting spatial immunity. The crypt epithelium is supported by a basement membrane and surrounded by the lamina propria, while the muscularis mucosae separates the mucosa from the submucosa. The lamina propria contains abundant T cells, B cells and plasma cells, macrophages, and dendritic cells, whereas intraepithelial lymphocytes occupy a distinct epithelial immune compartment. Studies of healthy human colon have demonstrated regional differences in immune-cell composition, and spatial mapping of the human intestine has identified organized mucosal immune neighborhoods, including plasma-cell-enriched niches within the lamina propria (16, 17). These findings establish a normal anatomical reference for interpreting immune-cell localization in primary CRC.

During invasive tumor growth, disruption of crypt architecture and invasion of surrounding tissues alter the relationships between epithelial, stromal, and immune compartments. Immune cells in adjacent nonmalignant lamina propria should therefore be distinguished from infiltrates within tumor-associated stroma and malignant epithelial nests. Spatial analyses have identified organized tumor-stroma boundaries in pMMR CRC that separate malignant regions from immune aggregates and stromal compartments (18). Consequently, an immune-rich mucosal or stromal region does not necessarily indicate effective lymphocyte penetration into malignant epithelial nests. Spatial assessments should account for adjacent nonmalignant mucosa, tumor-associated stroma, the invasive margin, and malignant epithelial compartments. This anatomical framework applies to primary colorectal tumors, while metastatic lesions require interpretation within their respective organ environments.

A schematic overview of the colonic tissue compartments and key cellular neighborhoods discussed in this review is provided in **Figure 1**.

**Figure 1:**
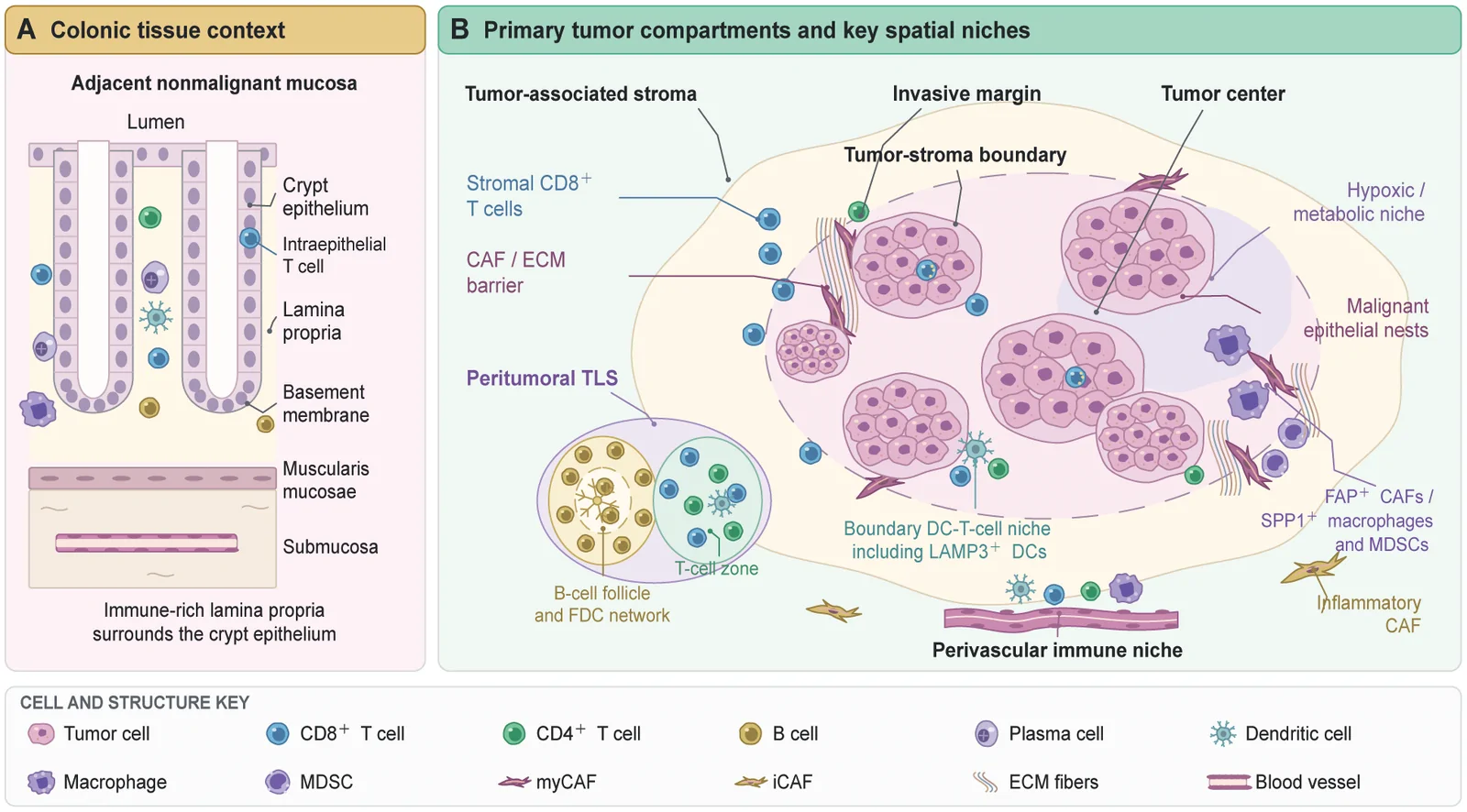
Overview of the spatial immune architecture in microsatellite-stable colorectal cancer. **(A)** Adjacent nonmalignant colonic mucosa provides an anatomical reference, showing crypt epithelium, basement membrane, immune-rich lamina propria, intraepithelial T cells, muscularis mucosae, and submucosa. **(B)** A conceptual map locates malignant epithelial nests relative to the tumor center, invasive margin, and tumor-associated stroma. Representative neighborhoods include stromal CD8^+^ T-cell accumulation, CAF-associated ECM barriers, DC-T-cell interfaces including LAMP3^+^ DCs, CAF-myeloid and hypoxic niches, perivascular immune neighborhoods, and a peritumoral TLS containing a B-cell follicle, an FDC network, and a T-cell zone. Cell abundances and spatial relationships are illustrative and context dependent; the depicted neighborhoods need not coexist in every MSS tumor. Supporting evidence includes broader CRC cohorts and preclinical models. CAF, cancer-associated fibroblast; CRC, colorectal cancer; DC, dendritic cell; ECM, extracellular matrix; FAP, fibroblast activation protein; FDC, follicular dendritic cell; iCAF, inflammatory CAF; LAMP3, lysosome-associated membrane glycoprotein 3; MDSC, myeloid-derived suppressor cell; MSS, microsatellite-stable; myCAF, myofibroblastic CAF; SPP1, secreted phosphoprotein 1; TLS, tertiary lymphoid structure.

### 2.1 Low neoantigen burden and insufficient T-cell priming

Most MSS CRCs are characterized by a low TMB, which limits the pool of potentially immunogenic neoantigens available for cross-presentation and cross-priming by BATF3-dependent type 1 conventional dendritic cells (cDC1s) (19, 20). Experimental expansion and activation of CD103^+^ dendritic cells enhanced treatment responses in melanoma models, supporting the importance of effective dendritic-cell function (21). WNT/β-catenin signaling is frequently activated in CRC, often through alterations in *APC* (adenomatous polyposis coli) (22). In melanoma models, tumor-intrinsic β-catenin signaling suppressed CCL4 expression through an ATF3-associated mechanism and impaired recruitment of BATF3-dependent dendritic cells (23). Pan-cancer associations between WNT/β-catenin activation and immune exclusion provide further rationale for investigating this pathway in MSS CRC (24, 25). However, these findings should not be interpreted as demonstrating the complete causal pathway in every human MSS tumor. Impaired antigen presentation and priming can contribute to sparse or functionally constrained T-cell infiltrates without implying the absence of all antigen-presenting cells.

### 2.2 WNT/β-catenin, TGF-β, and MAPK signaling: immune exclusion programs

Beyond cDC1 exclusion, β-catenin stabilization may also support the persistence of regulatory T cells, while tumor-cell WNT/β-catenin-Snail signaling can engage reciprocal crosstalk with macrophages, thereby reinforcing a tumor-promoting and potentially immunosuppressive microenvironment (26, 27). Transforming growth factor-β (TGF-β), frequently upregulated in consensus molecular subtype (CMS) 4 MSS tumors, can contribute to immune resistance through both stromal effects and direct suppression of cytotoxic T-cell programs (28). Experimental studies demonstrate SMAD-dependent transcriptional repression of cytolytic genes (29). Its effects on CAF activation, matrix remodeling, and spatial immune exclusion are discussed in Section 4.2. Alterations in the RAS–RAF–mitogen-activated protein kinase (MAPK) pathway are common in non-hypermutated CRC. Oncogenic KRAS and BRAF signaling can promote the production of proangiogenic and myeloid-recruiting mediators, including vascular endothelial growth factor A (VEGF-A) and CXCL8/interleukin-8 (IL-8). VEGF-A contributes to abnormal and immunologically anergic tumor vasculature, thereby reducing endothelial adhesion and chemokine signals required for T-cell trafficking, whereas IL-8 can recruit neutrophils, further reinforcing an immune-excluded microenvironment (22, 30, 31). Spatially, these signaling programs can contribute to dense stromal rims at the invasive margin, with CD8^+^ T cells arrested at the tumor-stroma boundary rather than penetrating malignant epithelial nests (18).

### 2.3 Myeloid suppressive cells and metabolic niches

Myeloid populations establish spatially organized immunosuppressive niches in MSS CRC. In primary CRC and liver metastases, secreted phosphoprotein 1 (SPP1)^+^ macrophages localize to hypoxic, necrotic, and desmoplastic regions and engage in spatial crosstalk with fibroblast activation protein (FAP)^+^ fibroblasts, in niches associated with limited lymphocyte access to malignant epithelial compartments (32, 33). Macrophages expressing triggering receptor expressed on myeloid cells 2 (TREM2) have also been implicated in immune suppression in experimental tumors. These myeloid states may impair T-cell function through complementary metabolic mechanisms, including arginase 1-dependent L-arginine depletion, adenosinergic signaling, and dysregulated lipid metabolism. However, direct evidence that all these pathways operate within the same SPP1^+^/TREM2^+^ spatial niche in MSS CRC remains limited (34–36). Myeloid-derived suppressor cells (MDSCs) can further suppress T-cell activity and interact with stromal and vascular compartments. Reviews propose that their distribution and plasticity contribute to immune-resistant architectures, but the precise relationship between MDSCs, tumor-associated macrophages, and malignant glands requires direct spatial validation in human MSS CRC (37, 38).

### 2.4 Abnormal vasculature and hypoxia

An early CRC study reported lower VEGF expression in neoplasms with microsatellite instability (39). VEGF-driven tumor angiogenesis can produce tortuous, hyperpermeable vessels with inadequate pericyte coverage. These abnormalities can impair perfusion and endothelial activation, limiting immune-cell adhesion and extravasation. Experimental studies show that, within hypoxic tumor regions, stabilization of hypoxia-inducible factor-1α (HIF-1α) activates a proangiogenic and immunosuppressive transcriptional program, directly inducing VEGF and PD-L1 expression, including PD-L1 on malignant and myeloid cells, while promoting galectin-9 expression indirectly through a HIF-1-TGF-β-SMAD3 signaling axis in cancer cells (40, 41). Hypoxic and highly glycolytic tumor regions accumulate lactic acid and develop extracellular acidosis, which disrupts lactate export and glycolytic metabolism in activated T cells, thereby suppressing their proliferation, cytokine production, and cytolytic activity (42). These vascular and metabolic abnormalities can jointly limit immune-cell access and effector function. Strategies for remodeling tumor vessels are discussed in Section 4.5.

### 2.5 Cancer-associated fibroblasts and extracellular matrix barriers

CAFs can contribute to immune resistance through ECM deposition and remodeling and the secretion of mediators that influence immune-cell recruitment (43–46). Stromal accumulation at malignant epithelial interfaces can limit lymphocyte access in pMMR CRC (18). The compartmental localization of CAF states, matrix architecture, and their effects on T-cell migration are examined in Sections 4.1–4.4.

In summary, immunotherapy resistance in MSS CRC arises from multiple, interconnected defects rather than from low immunogenicity alone. Insufficient T-cell priming is compounded by oncogenic immune-exclusion programs, suppressive myeloid niches, hypoxic and dysfunctional vasculature, and CAF-driven stromal barriers that collectively prevent effective T-cell recruitment, penetration, and function. These mechanisms converge spatially to generate immune-excluded microenvironments in which effector cells either fail to reach the tumor or remain confined outside malignant epithelial compartments. Thus, the resistance of MSS CRC to ICIs is best understood as a spatially organized ecosystem of tumor-intrinsic and microenvironmental barriers rather than a single molecular defect (**Figure 2**).

**Figure 2:**
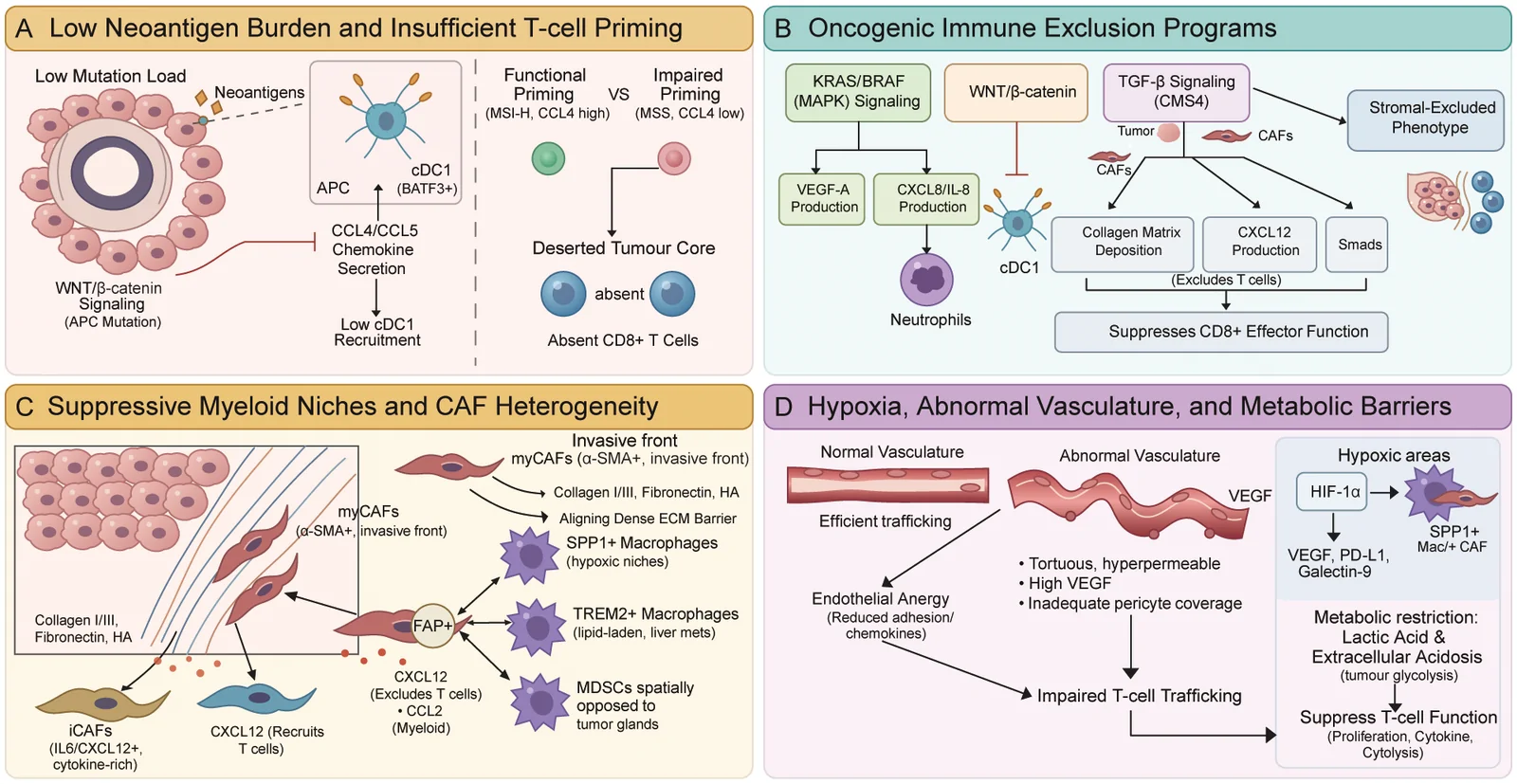
Biological basis of immunotherapy resistance in microsatellite-stable colorectal cancer. **(A)** Low neoantigen availability and impaired recruitment or function of type 1 conventional dendritic cells can compromise T-cell priming. Tumor-intrinsic WNT/β-catenin signaling provides an experimental framework for deficient dendritic-cell recruitment and immune exclusion. **(B)** Oncogenic KRAS/BRAF–MAPK, WNT/β-catenin, and TGF-β programs can promote myeloid recruitment, abnormal angiogenesis, and fibroblast-associated exclusion. Stromal remodeling limits access to tumor nests, while TGF-β signaling can also suppress cytotoxic T-cell function. **(C)** Matrix-producing fibroblasts and chemokine-rich fibroblast states contribute to stromal barriers and suppressive myeloid niches. SPP1^+^ and TREM2^+^ macrophage states and myeloid-derived suppressor cells represent distinct, potentially overlapping components of this environment. **(D)** Abnormal vasculature can impair immune-cell adhesion and extravasation. Hypoxia and extracellular acidosis further constrain T-cell function. Normal vessels are shown as a reference for efficient trafficking. APC, adenomatous polyposis coli when referring to the gene, or antigen-presenting cell when referring to a cell; BATF3, basic leucine zipper ATF-like transcription factor 3; CAF, cancer-associated fibroblast; cDC1, type 1 conventional dendritic cell; CMS4, consensus molecular subtype 4; ECM, extracellular matrix; FAP, fibroblast activation protein; HIF-1α, hypoxia-inducible factor-1α; iCAF, inflammatory CAF; MAPK, mitogen-activated protein kinase; MDSC, myeloid-derived suppressor cell; MSI-H, microsatellite instability-high; myCAF, myofibroblastic CAF; PD-L1, programmed death-ligand 1; SPP1, secreted phosphoprotein 1; TGF-β, transforming growth factor-β; TREM2, triggering receptor expressed on myeloid cells 2; VEGF, vascular endothelial growth factor; α-SMA, α-smooth muscle actin.

## 3 Spatial immune phenotypes in MSS CRC

The spatial distribution of immune cells within CRC tissue may provide information about prognosis and treatment response beyond overall cell counts. This section examines spatial immune phenotypes specifically in MSS CRC, emphasizing tissue-compartment-specific distributions and cell-cell proximity metrics. Prognostic associations concern outcome, whereas predictive evidence concerns differential benefit from a specified treatment. Correlations within ICI-treated cohorts alone do not establish ICI-specific prediction. **Table 1** summarizes the clinical studies discussed in this review, including cohort sizes, treatment regimens, tissue or spatial profiling approaches, clinical outcomes, and limitations of the reported biomarker associations.

**Table 1:** Key clinical studies and tissue or spatial correlates of immunotherapy in MSS/pMMR colorectal cancer.

Study and design	Population and sample size	Treatment regimen	Clinical outcome	Tissue or spatial profiling	Outcome association and evidence limits
REGONIVO Phase Ib, single arm Fukuoka et al., 2020 (8)	Refractory mCRC; 25 CRC patients (24 MSS, 1 MSI-H) within a 50-patient gastric/CRC study.	Regorafenib + nivolumab.	CRC cohort: ORR 36% (9/25); median PFS 7.9 months.	Tumor PD-L1 and TMB assessment; no validated spatial selection metric.	Initial activity in a small, single-arm cohort. The cited trial does not establish a spatial predictor of ICI benefit.
REGOMUNE Phase II, single arm Cousin et al., 2021 (9)	Refractory MSS mCRC; 48 enrolled, 43 efficacy-evaluable.	Regorafenib + avelumab.	No objective responses; median PFS 3.6 months and OS 10.8 months.	Multiplex IF and digital tumor/stroma segmentation of baseline and on-treatment biopsies.	High baseline intratumoral CD163+ macrophage density was associated with worse PFS/OS; increased on-treatment CD8+ infiltration with better outcomes. Single-arm density/outcome associations.
NICHE Exploratory neoadjuvant study Chalabi et al., 2020 (10)	Resectable early-stage colon cancer; 35 combination-evaluable tumors, including 15 pMMR.	Ipilimumab + nivolumab before surgery; pMMR group with or without celecoxib.	pMMR: pathological response in 4/15 (27%), including 3 MPRs.	Tumor immune profiling; baseline CD8+PD-1+ T-cell infiltration.	Baseline CD8+PD-1+ infiltration was associated with pMMR pathological response. Exploratory association in 15 tumors; not independently validated or directly transferable to mCRC.
AtezoTRIBE Randomized phase II Antoniotti et al., 2022; Moretto et al., 2023 (50, 82)	First-line mCRC, mixed MMR status; 218 randomized. Immunoscore-IC assessed in 157 specimens; pMMR subgroup analyzed.	FOLFOXIRI + bevacizumab, with or without atezolizumab.	Overall cohort: median PFS 13.1 vs 11.5 months with vs without atezolizumab.	Digital tissue analysis; Immunoscore-IC integrates CD8+/PD-L1+ densities and spatial proximity.	High Immunoscore-IC identified greater atezolizumab PFS benefit in pMMR tumors (interaction P = 0.006). Preliminary predictive evidence for a composite metric; prospective validation required.
MAYA Phase II, single arm; spatial follow-up Choo et al., 2026 (49)	MGMT-silenced MSS mCRC; 135 started TMZ, 33 received the triplet. COMET: 26 patients (15 triplet, 11 TMZ-only); GeoMx: 16 baseline samples.	TMZ priming, then TMZ + nivolumab + low-dose ipilimumab if disease was controlled.	Selected triplet cohort: ORR 45%; 8-month PFS rate 36%.	COMET sequential IF; GeoMx digital spatial profiling; Visium spatial transcriptomics in smaller subsets.	Benefit associated with activated or proliferating CD8+ cells in tumor and interface regions; CD8+ proximity to CAFs or endothelium was unfavorable. Selected correlative cohorts; no randomized ICI-specific validation.
Regorafenib + sintilimab Phase II, single arm Liu et al., 2025 (51)	Refractory MSS mCRC; 103 treated. Multiplex IHC subset: 30 patients (15 sensitive, 15 resistant).	Regorafenib + sintilimab.	ORR 21.4% (22/103); median PFS 4.1 months and OS 14.1 months.	Multiplex IHC; immune-cell density and distances to cytokeratin-positive tumor cells.	Sensitive tumors had more CD8+ T cells and shorter CD8+-tumor-cell distances. Exploratory association in a selected tissue subset; incremental ICI benefit not established.
C1QC+ macrophage-CD4+ niche cohort Correlative tissue study Zhang et al., 2025 (53)	16 MSS patients within a 25-patient mixed MSI/MSS imaging mass cytometry cohort.	Regorafenib + ICIs in the MSS subgroup.	Benefit defined as partial response or stable disease maintained for >6 months; not ORR alone.	Imaging mass cytometry; supporting spatial proteomics and transcriptomics.	Closer CD4+ T-cell-C1QC+ macrophage apposition was associated with benefit; greater CAF interposition with lack of benefit. Small cohort; no prospective predictive validation.
IMT009 + fruquintinib Phase Ib; conference abstract Ulahannan et al., 2026 (60)	Second- to fourth-line MSS CRC; 19 combination-treated patients selected for tissue CD161+ cells.	IMT009 (anti-CD161) + fruquintinib.	1 confirmed partial response; 3 additional patients remained on treatment for >6 months.	Central CD161 IHC for eligibility; multiplex imaging of pretreatment tissue and TLS.	CLEC2D/CD161-positive TLS presence/number and CXCL13 were associated with benefit. Hypothesis-generating abstract; spatial-analysis denominator not specified and predictive value unvalidated.
IMblaze370 Randomized phase III Eng et al., 2019 (83)	Previously treated mCRC, predominantly MSS; 363 randomized (183 combination, 90 atezolizumab, 90 regorafenib).	Atezolizumab + cobimetinib, atezolizumab alone, or regorafenib.	No OS advantage; combination vs regorafenib: median OS 8.87 vs 8.51 months, HR 1.00.	No clinically validated spatial selection assay established in the primary report.	Negative randomized efficacy result. Biological rationale alone does not establish clinical benefit or a spatial response predictor.
Botensilimab + balstilimab Phase I, single arm Bullock et al., 2024 (84)	Refractory MSS mCRC; 148 treated, 101 response-evaluable with >=6 months of follow-up.	Botensilimab + balstilimab.	ORR 17% (17/101); DCR 61%.	Clinical liver-metastasis subgroup analysis; no validated tissue spatial selection metric.	ORR 22% without active liver metastases vs 0% with active liver metastases. Organ-site association is exploratory and does not validate a spatial biomarker.
CCTG CO.26 Randomized phase II Chen et al., 2020 (85)	Refractory advanced CRC, predominantly MSS; 180 randomized, 179 treated.	Durvalumab + tremelimumab + BSC vs BSC alone.	Median OS 6.6 vs 4.1 months; P = 0.07 met prespecified alpha = 0.10. PFS not improved.	Baseline plasma cell-free DNA sequencing for MSI/TMB; nonspatial assay.	Exploratory plasma TMB association with OS benefit. No spatial response metric was validated.

BSC, best supportive care; CAF, cancer-associated fibroblast; C1QC, complement C1q C chain; CCTG, Canadian Cancer Trials Group; CRC, colorectal cancer; DCR, disease control rate; FOLFOXIRI, fluorouracil, leucovorin, oxaliplatin, and irinotecan; HR, hazard ratio; ICI, immune checkpoint inhibitor; IF, immunofluorescence; IHC, immunohistochemistry; mCRC, metastatic CRC; MGMT, O6-methylguanine-DNA methyltransferase; MMR, mismatch repair; MPR, major pathological response; MSI, microsatellite instability; MSI-H, MSI-high; MSS, microsatellite stable; ORR, objective response rate; OS, overall survival; PD-1, programmed cell death protein 1; PD-L1, programmed death-ligand 1; PFS, progression-free survival; pMMR, mismatch repair proficient; TLS, tertiary lymphoid structure; TMB, tumor mutational burden; TMZ, temozolomide.

Immune-desert tumors contain few T cells in both malignant epithelial compartments and tumor-associated stroma, including the invasive margin. This differs from immune-excluded tumors, in which T cells accumulate in stromal or peripheral regions but remain sparse within malignant epithelial nests; immune-inflamed tumors show intratumoral T-cell infiltration (14; **Figure 3**). Low neoantigen availability, inadequate dendritic-cell-mediated priming, and defective chemokine-mediated recruitment may contribute to an immune-desert pattern, as discussed in Section 2.1 (19, 20, 23). This mechanistic framework partly derives from preclinical and non-CRC studies and does not establish which process predominates in individual human MSS tumors. The designation reflects T-cell paucity rather than the absence of all immune cells. Assessment should cover multiple tissue regions and compartments because a small biopsy may miss localized immune-active niches; MSS-specific thresholds linking these phenotypes to ICI benefit remain unvalidated.

**Figure 3:**
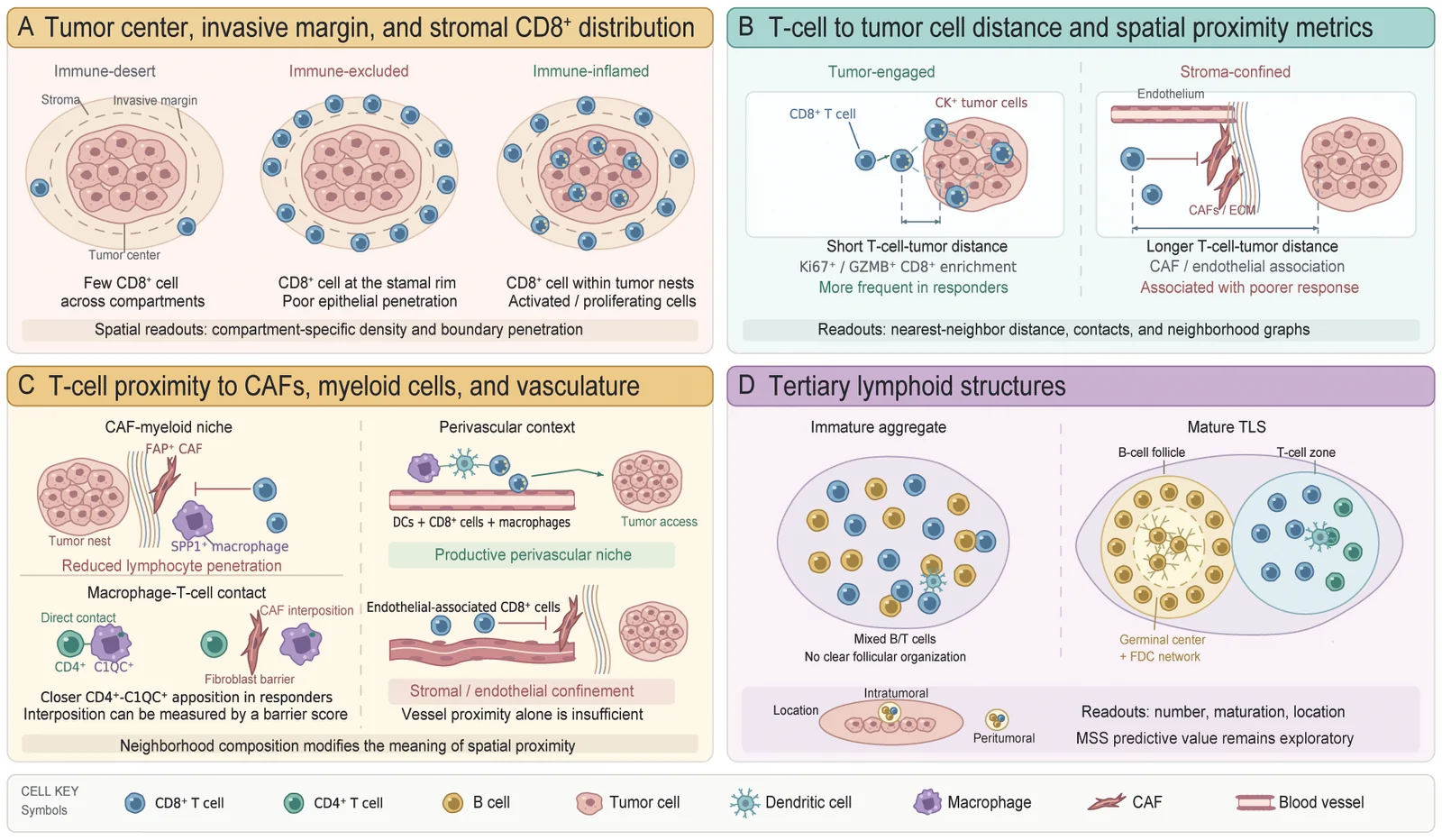
Spatial immune phenotypes in microsatellite-stable colorectal cancer. **(A)** Immune-desert, immune-excluded, and immune-inflamed patterns differ in the abundance and compartmental distribution of CD8^+^ T cells. In excluded tumors, lymphocytes accumulate in stromal regions or at the invasive margin while remaining sparse within malignant epithelial nests. **(B)** Distances between CD8^+^ T cells and cytokeratin-positive tumor cells capture immune access beyond overall cell density. Shorter distances and intratumoral proliferating or cytotoxic T cells have been associated with response in selected MSS CRC combination-treatment cohorts. **(C)** Cellular neighborhoods modify the interpretation of proximity. Fibroblast-associated macrophage niches may restrict lymphocyte access, whereas closer CD4^+^ T-cell–C1QC^+^ macrophage relationships have been associated with sustained clinical benefit in a small cohort. Productive perivascular immune niches are supported by preclinical models; proximity to vessels alone does not define a functional or dysfunctional state. **(D)** Tertiary lymphoid structures vary in organization, maturity, and location. Mature structures contain segregated B-cell follicles and T-cell zones, follicular dendritic-cell networks, and germinal-center features. These properties are candidate measures of local immune organization. CAF, cancer-associated fibroblast; C1QC, complement C1q C chain; CK, cytokeratin; DC, dendritic cell; ECM, extracellular matrix; FAP, fibroblast activation protein; FDC, follicular dendritic cell; GZMB, granzyme B; SPP1, secreted phosphoprotein 1; TLS, tertiary lymphoid structure.

### 3.1 Tumor center, invasive margin, and stromal CD8^+^ distribution

The consensus Immunoscore, internationally validated in 2,681 patients with stage I-III colon cancer across 14 centers in 13 countries, established that CD3^+^ and CD8^+^ T-cell densities in the tumor center and invasive margin provide prognostic information beyond conventional clinicopathological staging (47, 48). However, the conventional Immunoscore was developed primarily as a prognostic rather than an immunotherapy-predictive tool and does not fully capture the finer spatial relationships that may influence ICI sensitivity in MSS CRC (49). In the AtezoTRIBE randomized phase II trial of fluorouracil, leucovorin, oxaliplatin, and irinotecan (FOLFOXIRI) plus bevacizumab with or without atezolizumab, the Immunoscore-Immune Checkpoint (Immunoscore-IC) metric integrates CD8^+^ and PD-L1^+^ cell densities with their spatial proximity. An exploratory treatment-by-biomarker analysis suggested greater PFS benefit from adding atezolizumab in Immunoscore-IC-high pMMR tumors. This provides preliminary predictive evidence for the composite metric in this regimen, but does not validate proximity alone or establish generalizability across ICI regimens (50). Similarly, in the MAYA trial spatial profiling, lymphocyte proportions, particularly CD8^+^ Ki-67^+^ proliferating cytotoxic T cells, within stromal and tumor compartments, alongside macrophage composition at the tumor-stromal interface, were associated with response to temozolomide-primed dual checkpoint blockade. This analysis involved selected tumors with O6-methylguanine-DNA methyltransferase (MGMT) silencing; the spatial proteomic comparison of triplet-treated patients included 15 cases, limiting generalizability to unselected MSS mCRC (49). An alternative approach is to measure immune-cell penetration relative to an anatomically defined tumor-stroma boundary rather than to individual malignant cells. Spatial transcriptomic mapping of CRC relative to a tumor–stroma boundary showed that immune cells in pMMR tumors preferentially accumulated on the stromal side and declined discontinuously toward the tumor center, whereas CXCL13^+^ T cells, effector CD8^+^ T cells, and LAMP3^+^ dendritic cells were associated with more immune-permissive boundaries (18). These findings suggest that compartment-specific T-cell localization and activation provide information not captured by TMB alone.

### 3.2 T-cell to tumor cell distance and spatial proximity metrics

The physical distance between CD8^+^ T cells and malignant epithelial cells has been examined as an exploratory spatial correlate of treatment outcome. In a single-arm study of regorafenib plus sintilimab in MSS mCRC, multiplex immunohistochemistry (mIHC) showed that treatment-sensitive tumors had shorter distances between CD8^+^ T cells and cytokeratin-positive tumor cells than treatment-resistant tumors, alongside higher CD8^+^ T-cell densities (51). Beyond pairwise distance, graph- and neighborhood-based spatial analyses can characterize whether CD8^+^ T cells form cohesive intratumoral aggregates or remain dispersed within, or confined to, stromal compartments. In the selected MAYA cohort, response was associated with the compartment-specific enrichment of activated and proliferating CD8^+^ T cells within malignant epithelial regions and the tumor-stromal interface, whereas close spatial associations between CD8^+^ T cells and CAFs or endothelial cells were associated with poor response (49). Mechanistically, greater distances between T cells and malignant cells may reflect defective chemokine-mediated recruitment rather than a purely physical barrier. In CRC liver metastasis models, tumor-derived WNT11 suppressed CXCL10 and CCL4 expression, reduced CD8^+^ T-cell recruitment, and promoted resistance to anti-PD-1 therapy, providing a biological explanation for spatially distant or excluded T-cell patterns (52).

### 3.3 T-cell proximity to CAFs, myeloid cells, and vasculature

The cellular neighborhood context of T cells can influence their functional state. Spatial proteomic analyses using co-detection by indexing (CODEX) on CRC tissue microarrays (although not restricted to MSS disease) have identified coordinated cellular neighborhoods at the invasive front whose composition stratifies immune pressure and clinical outcome (12). A distinct mesenchymal-myeloid spatial circuit has subsequently been identified in CRC, in which SPP1^+^ macrophages colocalize with FAP^+^ fibroblasts around malignant epithelial regions. Related expression programs were examined across 14 independent CRC cohorts, with spatial transcriptomics and immunofluorescence supporting cellular colocalization in analyzed tissues. The corresponding niches were enriched in extracellular-matrix organization, collagen remodeling, hypoxia, and TGF-β-associated programs and were accompanied by reduced lymphocyte penetration into the tumor core (32). Although these cohorts were not specifically restricted to MSS/pMMR disease, the desmoplastic and immune-excluded architecture defined by FAP^+^ fibroblast-SPP1^+^ macrophage proximity is highly relevant to the stromal-dominant immune phenotype frequently observed in MSS CRC. Spatial relationships with myeloid cells also contribute to treatment-associated immune architecture. Imaging mass cytometry in a 16-patient MSS subgroup associated sustained clinical benefit with closer spatial apposition between CD4^+^ T cells and complement C1q C chain (C1QC)^+^ macrophages. Patients without benefit showed greater fibroblast interposition, quantified using a fibroblast barrier score. Benefit included stable disease or partial response lasting more than 6 months, rather than objective response alone (53).

Lysosome-associated membrane glycoprotein 3 (LAMP3)^+^ dendritic cells represent a mature, migratory DC state with immunoregulatory features (18). Mechanistic work in human and mouse lung cancer showed that type 1 and type 2 conventional DCs can acquire a shared maturation and regulatory program after tumor-antigen uptake, including CCR7, CD40, and PD-L1 expression (54). This illustrates how DC maturation can coexist with both T-cell stimulation and inhibitory signaling. Nomenclature varies across studies: LAMP3^+^ DCs should not automatically be equated with the circulating inflammatory DC3 subset defined by CD163/CD14 expression (55). Here, LAMP3^+^ DCs are discussed as a marker-defined functional state. In CRC, spatial profiling identified enrichment of LAMP3^+^ DCs at tumor-stroma boundaries and close juxtaposition with CXCL13^+^ CD4^+^ and CD8^+^ T cells. These DCs coexpressed PD-L1 and costimulatory molecules such as CD80/CD86, suggesting that their local interactions can combine activating and inhibitory signals. Boundary enrichment was greater in dMMR than pMMR tumors, and treatment-associated analyses linked this architecture to response mainly in dMMR CRC (18). These findings support examining LAMP3^+^ DC localization and T-cell neighborhoods alongside cell abundance. Their independent predictive value for ICI benefit in MSS CRC remains unvalidated, and observations from dMMR CRC require separate validation in MSS disease.

Vascular proximity represents an important component of the spatial immune context, although a fixed vessel-distance threshold associated with T-cell dysfunction has not yet been established in human MSS CRC. In preclinical colorectal tumor models, CD8^+^ T cells cluster with dendritic cells and macrophages near intratumoral blood vessels, forming perivascular immune niches that expand following effective immunotherapy and are associated with productive antitumor immune responses (56). In the MAYA cohort, responders showed enrichment of granzyme B-positive and proliferating CD8^+^ T cells within malignant epithelial regions and the tumor–stromal interface, whereas CD8^+^ T cells positioned relatively closer to endothelial cells or CAFs than to tumor cells were associated with poorer response (49).

### 3.4 Tertiary lymphoid structures

TLS are organized ectopic lymphoid aggregates containing B-cell follicles, T-cell zones, and mature dendritic cells, resembling secondary lymphoid organs. In CRC, TLS presence correlates with improved prognosis and enhanced immune surveillance (13). These prognostic associations do not establish ICI-specific prediction, and the predictive value of baseline TLS number for ICI benefit in MSS CRC remains unvalidated.

TLS are generally less abundant and less mature in MSS than in MSI-H CRC, although they are not uniformly absent from MSS tumors. Accordingly, TLS presence alone may have limited discriminatory value, whereas maturation state and spatial localization may provide more biologically and clinically informative measures (57, 58). Mature TLS, characterized by spatially segregated CD20^+^ B-cell follicles and CD3^+^ T-cell zones together with follicular dendritic-cell (FDC) networks and germinal-center features, have been studied as spatial correlates of antitumor immunity. In three independent cohorts of patients with solid tumors treated with anti-PD-1/PD-L1 therapy, the presence of mature TLS was associated with higher objective response rates and prolonged progression-free and overall survival independently of PD-L1 expression and intratumoral CD8^+^ T-cell density (59). These pan-tumor outcome associations require separate validation before mature TLS can be considered an ICI-specific predictive biomarker in MSS CRC.

Early clinical evidence further suggests that the functional composition of TLS may be relevant in selected MSS CRC subsets. A 2026 conference abstract from the phase 1b study of IMT009 plus fruquintinib in MSS CRC reported exploratory associations between clinical benefit and pretreatment CLEC2D/CD161-positive TLS features, including their presence and number, together with CXCL13-associated immune features (60). These findings arose from a small, selected cohort and remain hypothesis-generating; the abstract does not establish TLS maturity or compartment-specific CLEC2D/CD161 localization as validated predictors. Importantly, TLS formation may also be therapeutically inducible in otherwise immune-cold CRC. Preclinical studies demonstrate that enforced expression of the lymphoid-organizing cytokine tumor necrosis factor superfamily member 14 (LIGHT) can promote intratumoral lymphoid organization and T-cell recruitment, and LIGHT-driven induction of mature TLS has recently been demonstrated through activation of the LIGHT–herpesvirus entry mediator axis in CRC models (61). Consistently, in an MSS-like CRC liver metastasis model, LIGHT overexpression combined with cytotoxic T-lymphocyte-associated protein 4 (CTLA-4) blockade promoted lymphocyte homing, reversed T-cell exhaustion and myeloid suppression, and improved tumor control (62). Thus, while baseline TLS density may be low in MSS CRC, their treatment-induced emergence or maturation could serve as a dynamic spatial biomarker of immune reactivation. However, neither these model studies nor the early clinical observations establish TLS number or maturity as independently validated predictors of treatment response in MSS CRC.

Collectively, these studies indicate that the immune phenotype of MSS CRC cannot be adequately captured by lymphocyte abundance alone. Across selected treatment cohorts, favorable outcomes have been associated with a spatially permissive architecture characterized by activated and proliferating T cells penetrating malignant epithelial compartments, close apposition of lymphocytes to malignant or antigen-presenting cells, coordinated myeloid-lymphoid niches, and mature TLS in selected cases. Conversely, unfavorable outcomes have been associated with stromal confinement of T cells, fibroblast- and endothelial-associated exclusion, suppressive macrophage-CAF neighborhoods, and poorly organized or immature lymphoid aggregates. These observations support a conceptual continuum from immune-inflamed to immune-excluded and immune-desert states in MSS CRC, with treatment potentially shifting tumors between these spatial phenotypes (**Figure 3**).

## 4 Stromal and extracellular-matrix barriers

Building on the mechanisms outlined in Section 2, this section examines the compartmental localization of CAF states, the composition and organization of the ECM, and their effects on T-cell access and migration. It also considers how vascular remodeling may alter these spatial barriers.

### 4.1 CAF subpopulations and their spatial localization

Single-cell and spatial analyses have identified heterogeneous CAF states at CRC tumor–stroma boundaries (18, 32). Commonly described states include myofibroblastic CAFs (myCAFs), with α-smooth muscle actin (α-SMA; encoded by ACTA2) and matrix-associated programs, and inflammatory CAFs (iCAFs), with cytokine- and chemokine-rich programs. Matrix-associated programs can also include type I collagen (*COL1A1*/*COL1A2*), type III collagen (*COL3A1*), and fibronectin (*FN1*). The myCAF–iCAF framework was developed in part in pancreatic cancer and should not be assumed to define a universal spatial hierarchy in MSS CRC (63). Experimental studies show that CAF contractility can remodel and align fibronectin, guiding cancer-cell migration; direct effects on T-cell migration require separate evidence (64). In CRC, ECM-producing CAF populations preferentially localize to desmoplastic tumor-stroma boundaries, where collagen-rich matrix programs are associated with restricted penetration of lymphocytes into malignant epithelial compartments (18). The matricellular protein thrombospondin-2 (THBS2), which is highly expressed by matrix CAFs at the tumor front in fibrotic, predominantly MSS/CMS4 CRC, has been implicated as a regulator of immune exclusion in CRC models. Orthotopic implantation of CMS4-like tumor organoids into mice with global or fibroblast-specific *Thbs2* deletion markedly increased intratumoral CD8^+^ T-cell infiltration and tumor-cell apoptosis (65). iCAFs display cytokine- and chemokine-rich programs characterized by IL6 and CXCL12 expression but their localization relative to matrix-producing CAFs varies with tumor context. CAF-derived CXCL12 may contribute to T-cell exclusion, whereas CCL2 primarily reinforces immunosuppression through recruitment of suppressive myeloid cells (43, 44, 63). Inflammatory fibroblasts have also been implicated in resistance to neoadjuvant therapy in rectal cancer, although this is not equivalent to evidence of ICI-specific resistance (66). Studies of fibroblast heterogeneity and spatial proteomic classification in breast cancer further illustrate the diversity of CAF states but do not directly validate the same states in MSS CRC (67, 68). These observations raise the possibility that the relative balance and spatial distribution of myCAF versus iCAF states, rather than total CAF abundance alone, may provide a more refined measure of stromal immune exclusion in MSS CRC.

### 4.2 TGF-β-associated stromal remodeling

TGF-β signaling is a central driver of stromal activation and immune exclusion in mesenchymal, CMS4-like CRC. Stromal TGF-β-response programs are strongly enriched in poor-prognosis CRC and are largely attributable to CAFs rather than malignant epithelial cells (45). Clinical observations in urothelial cancer and experimental studies link fibroblast TGF-β signaling to collagen-rich stroma and T-cell exclusion (69). In genetically engineered CRC models recapitulating key features of MSS disease, a TGF-β-activated stroma was associated with T-cell exclusion and resistance to PD-1/PD-L1 blockade, whereas TGF-β inhibition restored cytotoxic T-cell infiltration and sensitized established metastases to checkpoint blockade (70). TGF-β signaling also promotes metastatic niche formation through long-range stromal communication. In patient-derived CRC CAFs, TGF-β stimulation induces IL-6-family cytokines, particularly IL-6 and IL-11, which activate glycoprotein 130 (gp130)-dependent Janus kinase/signal transducer and activator of transcription (JAK/STAT) signaling in hepatocytes and induce myeloid chemoattractants including serum amyloid A1 (SAA1) and CXCL5, thereby promoting neutrophil recruitment and establishment of a pro-metastatic hepatic microenvironment (71). Locally, TGF-β-driven stromal activation is closely linked to extracellular-matrix remodeling and desmoplasia. Rather than acting solely as a passive physical barrier, this fibrotic program reorganizes the tumor microenvironment in ways that restrict productive T-cell infiltration and reinforce immunosuppressive cellular interactions. Recent mechanistic work in advanced CRC models further showed that TGF-β directly limits recruitment and accumulation of peripheral memory CD8^+^ T cells while simultaneously reprogramming tumor-associated macrophages toward an SPP1^+^ immunosuppressive state. Macrophage-derived SPP1 subsequently promotes collagen deposition and accumulation of fibroblasts and macrophages, establishing a coupled stromal-myeloid barrier that limits CD8^+^ T-cell proliferation and reduces the efficacy of PD-L1 blockade (72). Together, these findings position TGF-β not simply as a fibroblast-activating cytokine but as an organizer of spatial immune exclusion in MSS-like CRC, coordinating CAF activation, extracellular-matrix remodeling, impaired T-cell recruitment, and suppressive macrophage states. The clinical value of TGF-β-directed combinations and related spatial biomarkers in MSS CRC remains to be prospectively established.

### 4.3 ECM components: collagen, hyaluronan, and versican

The ECM of CRC is extensively remodeled and contains collagens, glycoproteins, proteoglycans, and matricellular proteins. In mesenchymal/CMS4-like tumors, collagen-rich stromal programs are particularly prominent and contribute to immune exclusion. The effects of collagen density, fiber alignment, and remodeling on T-cell migration and function are examined in Section 4.4. Hyaluronan (HA) is another major stromal component associated with mesenchymal CRC. HA accumulation promotes extensive remodeling of the tumor microenvironment, although its effects on vascular compression and interstitial pressure have been more directly established in other desmoplastic tumors (73). Versican also contributes to immune regulation in CRC. Proteolytic processing of versican generates versikine, which promotes BATF3-dependent dendritic-cell differentiation and is associated with increased CD8^+^ T-cell infiltration (74).

### 4.4 ECM density and T-cell migration

ECM density and architecture can strongly influence T-cell trafficking within solid tumors. Live imaging of human lung tumor slices demonstrated that T cells migrate efficiently through loose collagen- and fibronectin-rich regions but show restricted motility in dense matrix areas. Aligned stromal fibers can further redirect T-cell movement along the tumor-stroma boundary and limit entry into malignant epithelial nests (75). Collagen density can also directly impair T-cell function. In three-dimensional collagen matrices, high collagen density reduced T-cell proliferation and cytotoxic activity, although these findings were derived largely from experimental and non-CRC systems rather than human MSS CRC (76). More directly in colon cancer, increased collagen deposition and fiber alignment driven by G protein-coupled receptor 4 (GPR4)–lysyl oxidase-like 2 (LOXL2) signaling in preclinical models were associated with reduced intratumoral CD8^+^ T-cell infiltration, whereas inhibition of collagen remodeling restored T-cell penetration and enhanced PD-L1 blockade efficacy (77). Beyond its physical effects, collagen can also transmit inhibitory signals to T cells. Studies mainly in lung cancer and other non-CRC systems show that collagen-rich tumors can promote CD8^+^ T-cell dysfunction through leukocyte-associated immunoglobulin-like receptor 1 (LAIR1)-dependent signaling, linking ECM composition to immunotherapy resistance independently of simple steric exclusion (78).

Together, collagen abundance, organization, and mechanical properties represent complementary features of the stromal barrier, although standardized quantitative thresholds for these parameters have not yet been validated as predictive biomarkers in MSS CRC.

### 4.5 Vascular normalization: breaking spatial exclusion

Anti-angiogenic therapy can transiently remodel abnormal tumor vessels, improve perfusion, and create a more permissive microenvironment for immune-cell infiltration (79). Vascular-normalizing doses of VEGF/VEGF receptor (VEGFR) blockade have also been shown in preclinical tumor models to enhance T-cell-dependent antitumor immunity (80). In mouse CRC models, vascular normalization was associated with tumor regression during PD-L1-based combination therapy (81).

In MSS CRC, regorafenib provides both anti-angiogenic and immunomodulatory activity through inhibition of VEGFRs and other stromal and immune-related kinases. In a single-arm phase II study of 103 patients with refractory MSS mCRC, regorafenib plus sintilimab achieved an ORR of 21.4% and median overall survival (OS) of 14.1 months, although the single-arm design does not establish the incremental benefit of adding sintilimab to regorafenib. In the tissue subset described above, treatment-sensitive tumors had higher baseline CD8^+^ T-cell density and shorter CD8^+^ T-cell-to-tumor-cell distances than resistant tumors, indicating that a pre-existing spatially permissive immune architecture may favor response to anti-angiogenic-ICI combinations. However, vascular normalization itself was not directly measured in this study (51). The randomized phase II AtezoTRIBE trial showed that adding atezolizumab to FOLFOXIRI plus bevacizumab improved PFS in metastatic CRC, with subsequent biomarker analyses indicating greater benefit in pMMR tumors with immune-enriched Immunoscore-IC profiles (50, 82).

These findings support vascular and immune remodeling as a promising strategy for overcoming spatial immune exclusion, while the optimal dose, treatment sequence, and spatial biomarkers for selecting MSS CRC patients remain to be prospectively defined.

Collectively, the stroma in MSS CRC functions as an active spatial barrier that integrates CAF heterogeneity, TGF-β signaling, ECM remodeling, and vascular dysfunction to restrict antitumor immunity. Matrix-producing CAF states and TGF-β-driven stromal-myeloid programs promote collagen-rich, immune-excluded niches, while ECM composition and architecture further constrain T-cell migration and function. Importantly, these barriers are potentially reversible: targeting TGF-β or ECM-remodeling pathways and combining vascular-modulating agents with ICIs may convert selected excluded tumors into more immune-permissive states (**Figure 4**). However, reliable biomarkers for identifying patients most likely to benefit from such strategies remain lacking, and quantitative thresholds for CAF states, matrix architecture, and vascular features have not been standardized in MSS CRC.

**Figure 4:**
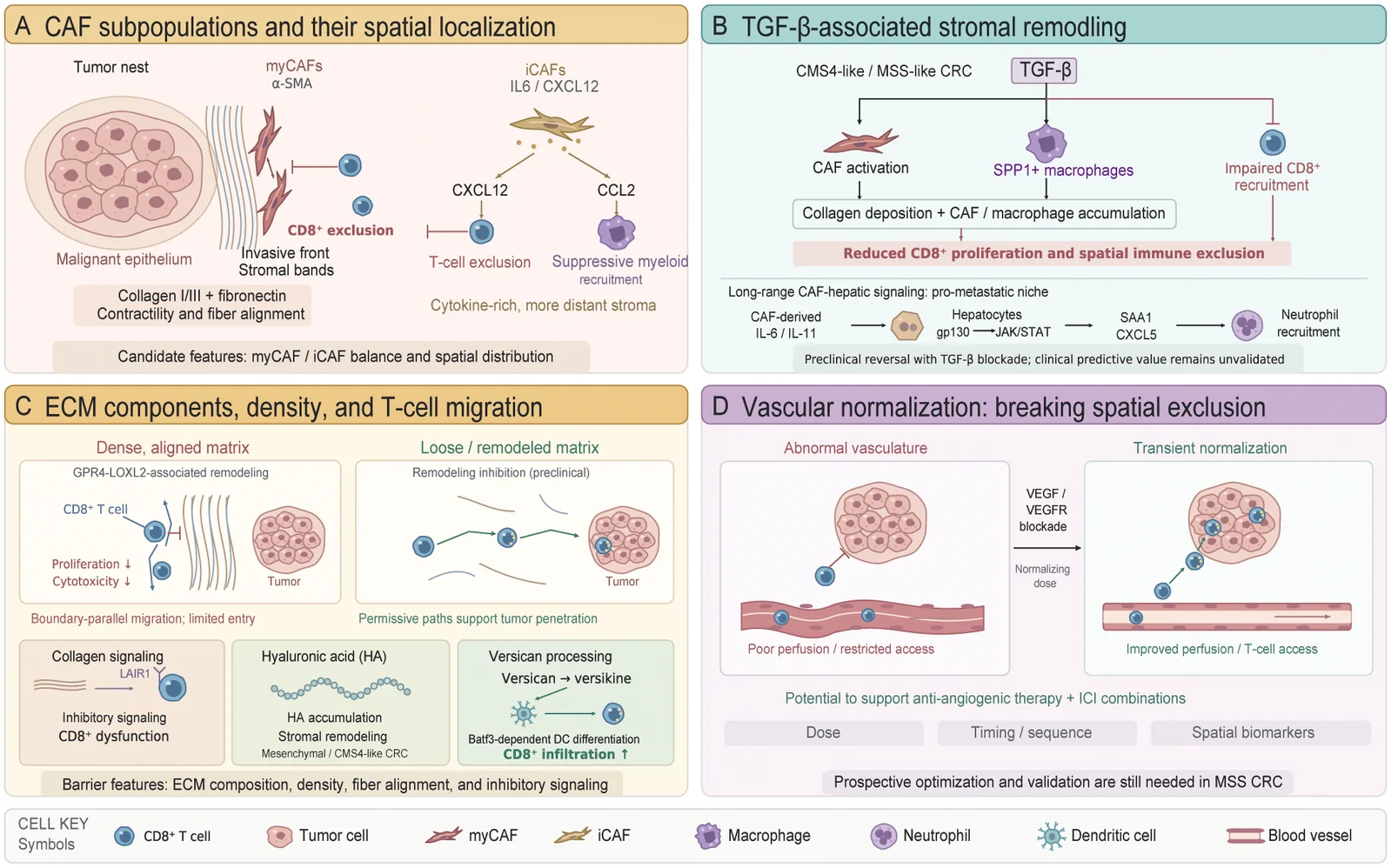
Stromal and extracellular-matrix barriers in microsatellite-stable colorectal cancer. **(A)** Representative fibroblast states contribute complementary matrix-producing and inflammatory programs. Their distribution and secreted factors can shape immune-cell recruitment and confinement, although myCAF–iCAF localization is context-dependent. **(B)** TGF-β coordinates fibroblast activation, extracellular matrix deposition, impaired CD8^+^ T-cell recruitment, and suppressive macrophage programs. The hepatic branch illustrates long-range signaling through fibroblast-derived IL-6-family cytokines and hepatocyte gp130–JAK/STAT responses that can promote a pre-metastatic niche. **(C)** ECM composition and density jointly influence T-cell movement and function. Collagen deposition and alignment can restrict penetration or redirect migration along stromal boundaries. GPR4–LOXL2-dependent remodeling is supported by preclinical colon cancer studies, whereas collagen–LAIR1 signaling is supported mainly by studies in other tumor types. Hyaluronan contributes to stromal remodeling, and versican processing can generate versikine, which promotes BATF3-dependent dendritic-cell differentiation. **(D)** Abnormal vessels and hypoxia can limit immune access. Appropriately dosed vascular-modulating treatment may create a transient normalization window that improves perfusion and immune trafficking. BATF3, basic leucine zipper ATF-like transcription factor 3; CAF, cancer-associated fibroblast; CMS4, consensus molecular subtype 4; ECM, extracellular matrix; GPR4, G protein-coupled receptor 4; HA, hyaluronan; iCAF, inflammatory CAF; IL, interleukin; JAK, Janus kinase; LAIR1, leukocyte-associated immunoglobulin-like receptor 1; LOXL2, lysyl oxidase-like 2; myCAF, myofibroblastic CAF; SAA1, serum amyloid A1; SPP1, secreted phosphoprotein 1; STAT, signal transducer and activator of transcription; TGF-β, transforming growth factor-β; VEGF, vascular endothelial growth factor; VEGFR, VEGF receptor; α-SMA, α-smooth muscle actin.

## 5 Discussion and future perspectives

The evidence reviewed provides three main insights into the questions raised in the Introduction. First, inadequate T-cell priming, oncogenic immune-exclusion programs, suppressive myeloid niches, and vascular or metabolic dysfunction can jointly establish spatial architectures that limit T-cell recruitment, access to malignant cells, and effector function. Second, enrichment of activated or proliferating T cells within malignant epithelial compartments, shorter T-cell–tumor-cell distances, and selected immune-cell neighborhoods have been associated with favorable outcomes in defined combination-treatment cohorts (49–51, 53). These associations remain exploratory and treatment-specific. They do not establish spatial features as clinically validated predictors of ICI benefit. Third, CAF-associated matrix deposition, increased collagen density, and fiber alignment can confine T cells to stromal regions or redirect their migration along tumor–stroma boundaries, limiting penetration into malignant epithelial nests. Matrix-associated inhibitory signaling may additionally impair T-cell function (18, 75–78). The causal evidence for some of these mechanisms derives from preclinical or non-CRC models and requires validation in human MSS CRC. Together, these findings emphasize that immune-cell localization and functional context are central to understanding spatial resistance, while their potential clinical applications require further validation.

Following limited activity with single-agent ICIs, combination strategies including chemo-immunotherapy, anti-angiogenic plus ICI, dual checkpoint blockade, and hypermutation priming have yielded higher response rates in selected populations. Although these advances have outpaced biomarker development, reliable tools are still lacking to identify which patients with MSS CRC are most likely to benefit from immunotherapy. These results are not uniform: the phase III IMblaze370 trial did not demonstrate an overall survival advantage for atezolizumab with or without cobimetinib over regorafenib in previously treated mCRC (83). Negative randomized studies caution against inferring clinical efficacy from mechanistic plausibility alone.

Dual checkpoint blockade has also shown activity in refractory MSS mCRC. In the phase I study of botensilimab, an Fc-enhanced anti-CTLA-4 antibody, plus balstilimab, an anti-PD-1 antibody, 148 patients were treated and 101 were response evaluable with at least 6 months of follow-up. ORR was 17% (17/101), with a disease control rate of 61%. In exploratory analyses, responses occurred in patients without active liver metastases (17/77; 22%), whereas none were observed among 24 patients with active liver metastases (84). This difference suggests that metastatic organ context may influence treatment activity, but the nonrandomized design and subgroup selection preclude causal conclusions or establishing a validated spatial biomarker. The randomized phase II Canadian Cancer Trials Group CO.26 study compared durvalumab (anti-PD-L1) plus tremelimumab (anti-CTLA-4) and best supportive care with best supportive care alone in 180 patients with refractory advanced CRC. Median OS was 6.6 versus 4.1 months (hazard ratio, 0.72; 90% confidence interval, 0.54–0.97; P = 0.07), meeting the trial’s prespecified two-sided 0.10 significance threshold. PFS was not improved, and grade 3 or higher adverse events were more frequent with immunotherapy. The MSS subgroup showed an OS signal, while the association between higher plasma TMB and greater benefit was exploratory (85). Together, these trials support further study of combination immunotherapy in selected MSS populations, without establishing a spatial predictor for routine treatment selection.

Interpretation of spatial features also requires attention to the heterogeneity of MSS/pMMR CRC. CMS1 is enriched for MSI and immune activation, so its immune features should not be generalized to MSS disease; CMS2 and CMS3 are characterized by canonical WNT/MYC and metabolic programs, respectively, whereas CMS4 is associated with stromal invasion, TGF-β activation, and angiogenesis (28). These bulk molecular categories provide biological context but do not directly specify local T-cell penetration or cellular neighborhoods. RAS/BRAF mutation status and right- versus left-sided primary location should likewise be considered as stratification factors. Evidence linking these strata to prospectively validated spatial predictors of ICI benefit in MSS CRC remains limited, and neither mutation status nor sidedness should be assumed to determine an immune-inflamed, immune-excluded, or immune-desert architecture.

Metastatic-site differences further limit extrapolation of spatial measurements. Matched single-cell and spatial profiling of primary CRC and liver metastases identified spatial reprogramming of suppressive macrophages and changes associated with neoadjuvant chemotherapy (86). Together with the macrophage–fibroblast networks described in MSS liver metastases (33), these findings indicate that metastatic architecture must be interpreted within its organ and treatment context. However, these tissue studies do not validate primary–metastatic interchangeability or ICI-specific predictive value. Spatial metrics measured in the primary tumor should therefore not be assumed to represent liver or other metastases, and conclusions from liver lesions should not automatically be transferred to lung or peritoneal disease. Studies should record the sampled organ, tissue compartment, and treatment timing and, where feasible, compare matched primary and metastatic samples.

Spatial biomarkers should complement molecular and clinical variables. Existing studies span early-stage pMMR colon cancer, refractory MSS mCRC, and treatment-specific populations, and their findings cannot be assumed to transfer across regimens. Most spatial analyses are exploratory, with limited sample sizes and selection based on tissue availability. A tumor with low overall CD8^+^ T-cell density may contain localized immune-active niches, whereas a tumor with high CD8^+^ density may show stromal confinement that restricts access to malignant cells. These observations support evaluating whether spatial features add prognostic information or identify differential benefit from ICI-containing regimens beyond molecular and clinical variables. Their ICI-specific predictive value requires independent prospective validation.

Future studies should address five questions: (i) Which immune cells are present? T-cell proliferation (Ki-67), cytotoxic potential (granzyme B), activation or dysfunction states, differentiation, and clonality should be considered alongside cell density. PD-1 expression alone does not distinguish activation from dysfunction. (ii) Where are they located? Tumor center, invasive margin, stromal bands, perivascular niches, or TLS? (iii) Which cells are they adjacent to? Tumor cells, CAFs, macrophages, or vessels? (iv) How do these spatial relationships change with therapy? Does treatment convert excluded to inflamed phenotypes, induce TLS maturation, normalize vasculature, or deplete suppressive myeloid niches? (v) Can these spatial signatures be replicated, validated, and standardized across independent cohorts, clinical trials, and technology platforms?

Spatial profiling platforms offer complementary information. Multiplex immunohistochemistry and immunofluorescence measure prespecified protein panels within preserved tissue architecture and can build on existing pathology workflows, but quantification depends on staining quality and cell segmentation. Spatial transcriptomics adds broad gene-expression context for identifying immune programs, although per-cell signal assignment depends on RNA sensitivity, spatial resolution, and segmentation or deconvolution (87, 88). CODEX increases protein multiplexing through cyclic fluorescence imaging, but antibody performance and image alignment require quality control (12). Imaging mass cytometry uses metal-tagged antibodies to avoid fluorescence-based spectral overlap, but requires specialized instrumentation and ablates the imaged tissue (89). Thus, protein imaging is well suited to cell phenotyping and proximity metrics, whereas RNA-based profiling adds discovery of local molecular programs; platform choice and cross-validation should match the intended spatial measure. Clonality generally requires T-cell receptor information in addition to spatial phenotyping. A single tissue section provides a local snapshot, and two-dimensional proximity does not by itself establish functional interaction. The challenge lies in standardization, validation, and clinical integration. We advocate for prospective trials in MSS CRC to incorporate prespecified spatial tissue sampling when feasible, centralized analysis, longitudinal on-treatment assessment, and correlation with circulating tumor DNA and radiographic endpoints. Prospective studies should test whether spatial measures improve treatment selection beyond molecular and clinical variables, helping identify patients with MSS mCRC who benefit from defined immunotherapy combinations.
