# Supplementary material for: Spatial immune architecture and immunotherapy resistance in microsatellite-stable colorectal cancer
Source: Front Immunol. 2026 Oct 26;17:1998498. doi: 10.3389/fimmu.2026.1998498 (PMC13746241; doi:10.3389/fimmu.2026.1998498)
Supplement: Supplementary file 1 [file Table1.docx]

# Supplementary Table S1. Glossary of abbreviations and molecular symbols used in the manuscript, with drug targets and clinical trial descriptions.

## Abbreviations and molecular symbols

| **Abbreviation or symbol** | **Full name or explanation** |
| --- | --- |
| ACTA2 | Actin alpha 2, smooth muscle; the gene encoding α-SMA |
| AI | Artificial intelligence |
| α-SMA | Alpha-smooth muscle actin |
| APC | Adenomatous polyposis coli when referring to the gene; antigen-presenting cell when referring to a cell |
| ATF3 | Activating transcription factor 3 |
| BATF3 | Basic leucine zipper ATF-like transcription factor 3 |
| BRAF | B-Raf proto-oncogene, serine/threonine kinase |
| C1QC | Complement C1q C chain |
| CAF | Cancer-associated fibroblast |
| CCL2 | C-C motif chemokine ligand 2 |
| CCL4 | C-C motif chemokine ligand 4 |
| CCR7 | C-C motif chemokine receptor 7 |
| CD3 | Cluster of differentiation 3 |
| CD4 | Cluster of differentiation 4 |
| CD8 | Cluster of differentiation 8 |
| CD14 | Cluster of differentiation 14 |
| CD20 | Cluster of differentiation 20 |
| CD40 | Cluster of differentiation 40 |
| CD80 | Cluster of differentiation 80 |
| CD86 | Cluster of differentiation 86 |
| CD103 | Cluster of differentiation 103 |
| CD161 | Cluster of differentiation 161; killer cell lectin-like receptor B1 |
| CD163 | Cluster of differentiation 163 |
| cDC1 | Type 1 conventional dendritic cell |
| CK | Cytokeratin |
| CLEC2D | C-type lectin domain family 2 member D |
| CMS | Consensus molecular subtype |
| CMS4 | Consensus molecular subtype 4 |
| CODEX | Co-detection by indexing |
| COL1A1 | Collagen type I alpha 1 chain |
| COL1A2 | Collagen type I alpha 2 chain |
| COL3A1 | Collagen type III alpha 1 chain |
| CRC | Colorectal cancer |
| CTLA-4 | Cytotoxic T-lymphocyte-associated protein 4 |
| CXCL5 | C-X-C motif chemokine ligand 5 |
| CXCL8 | C-X-C motif chemokine ligand 8; also known as IL-8 |
| CXCL10 | C-X-C motif chemokine ligand 10 |
| CXCL12 | C-X-C motif chemokine ligand 12 |
| CXCL13 | C-X-C motif chemokine ligand 13 |
| DC | Dendritic cell |
| DC3 | Dendritic cell subset 3; the circulating inflammatory subset discussed in the manuscript |
| dMMR | Mismatch repair-deficient |
| DNA | Deoxyribonucleic acid |
| ECM | Extracellular matrix |
| FAP | Fibroblast activation protein |
| Fc | Fragment crystallizable region of an antibody |
| FDC | Follicular dendritic cell |
| FN1 | Fibronectin 1 |
| FOLFOXIRI | Folinic acid (leucovorin), fluorouracil, oxaliplatin, and irinotecan chemotherapy regimen |
| gp130 | Glycoprotein 130 |
| GPR4 | G protein-coupled receptor 4 |
| GZMB | Granzyme B |
| HA | Hyaluronan |
| HIF-1 | Hypoxia-inducible factor 1 |
| HIF-1α | Hypoxia-inducible factor 1 alpha |
| HR | Hazard ratio |
| iCAF | Inflammatory cancer-associated fibroblast |
| ICI | Immune checkpoint inhibitor |
| IL | Interleukin |
| IL-6 / IL6 | Interleukin 6; IL6 denotes the gene symbol |
| IL-8 | Interleukin 8 |
| IL-11 | Interleukin 11 |
| Immunoscore-IC | Immunoscore-Immune Checkpoint |
| JAK | Janus kinase |
| Ki-67 | Ki-67 proliferation-associated nuclear antigen |
| KRAS | K-Ras proto-oncogene, GTPase; historically named Kirsten rat sarcoma viral oncogene homolog |
| LAIR1 | Leukocyte-associated immunoglobulin-like receptor 1 |
| LAMP3 | Lysosome-associated membrane glycoprotein 3 |
| LIGHT | Tumor necrosis factor superfamily member 14 |
| LOXL2 | Lysyl oxidase-like 2 |
| MAPK | Mitogen-activated protein kinase |
| mCRC | Metastatic colorectal cancer |
| MDSC | Myeloid-derived suppressor cell |
| MGMT | O⁶-methylguanine-DNA methyltransferase |
| mIHC | Multiplex immunohistochemistry |
| MSI-H | Microsatellite instability-high |
| MSS | Microsatellite-stable |
| myCAF | Myofibroblastic cancer-associated fibroblast |
| ORR | Objective response rate |
| OS | Overall survival |
| PD-1 | Programmed cell death protein 1 |
| PD-L1 | Programmed death-ligand 1 |
| PFS | Progression-free survival |
| pMMR | Mismatch repair-proficient |
| RAF | Rapidly accelerated fibrosarcoma; the RAF family of serine/threonine kinases |
| RAS | Rat sarcoma; the RAS family of small GTPases |
| SAA1 | Serum amyloid A1 |
| SMAD | SMAD family of intracellular signal transducers |
| SMAD3 | SMAD family member 3 |
| SPP1 | Secreted phosphoprotein 1 |
| STAT | Signal transducer and activator of transcription |
| TGF-β | Transforming growth factor beta |
| THBS2 / Thbs2 | Thrombospondin 2; human and mouse gene symbols, respectively |
| TLS | Tertiary lymphoid structure |
| TMB | Tumor mutational burden |
| TREM2 | Triggering receptor expressed on myeloid cells 2 |
| VEGF | Vascular endothelial growth factor |
| VEGF-A | Vascular endothelial growth factor A |
| VEGFR | Vascular endothelial growth factor receptor |
| WNT | Wnt family of secreted signaling proteins; the name derives from Wingless and Int-1 |
| WNT11 | Wnt family member 11 |

## Drugs and principal targets or mechanisms

| **Drug** | **Principal target or mechanism** | **Drug class or action** |
| --- | --- | --- |
| Atezolizumab | PD-L1 | Monoclonal antibody |
| Avelumab | PD-L1 | Monoclonal antibody; used in REGOMUNE |
| Balstilimab | PD-1 | Monoclonal antibody |
| Bevacizumab | VEGF-A | Monoclonal antibody that inhibits angiogenic signaling |
| Botensilimab | CTLA-4 | Fc-enhanced monoclonal antibody |
| Cobimetinib | Mitogen-activated protein kinase kinases 1 and 2 (MEK1/2) | Small-molecule kinase inhibitor |
| Durvalumab | PD-L1 | Monoclonal antibody |
| Fluorouracil | Thymidylate synthase and nucleic acid metabolism | Fluoropyrimidine antimetabolite |
| Fruquintinib | VEGFR1, VEGFR2, and VEGFR3 | Small-molecule kinase inhibitor |
| IMT-009 (IMT009) | CD161; blocks its interaction with CLEC2D | Monoclonal antibody |
| Ipilimumab | CTLA-4 | Monoclonal antibody |
| Irinotecan | Topoisomerase I | Topoisomerase inhibitor acting through an active metabolite |
| Leucovorin | Reduced folate cofactor | Enhances fluorouracil-mediated inhibition of thymidylate synthase |
| Nivolumab | PD-1 | Monoclonal antibody |
| Oxaliplatin | DNA | Platinum compound that forms DNA crosslinks |
| Pembrolizumab | PD-1 | Monoclonal antibody |
| Regorafenib | Multiple kinases, including VEGFRs and RAF kinases | Small-molecule multikinase inhibitor |
| Sintilimab | PD-1 | Monoclonal antibody |
| Temozolomide | DNA | Alkylating agent that methylates DNA |
| Tremelimumab | CTLA-4 | Monoclonal antibody |

## Clinical trial names

| **Trial name or code** | **Treatment context** |
| --- | --- |
| AtezoTRIBE | FOLFOXIRI plus bevacizumab, with or without atezolizumab, in metastatic CRC |
| CO.26 | Canadian Cancer Trials Group study of durvalumab plus tremelimumab and best supportive care versus best supportive care alone in refractory advanced CRC |
| IMblaze370 | Atezolizumab with or without cobimetinib versus regorafenib in previously treated metastatic CRC |
| KEYNOTE-177 | Pembrolizumab versus chemotherapy as first-line treatment for MSI-H/dMMR metastatic CRC |
| MAYA | Temozolomide priming followed by ipilimumab plus nivolumab in selected MGMT-silenced MSS metastatic CRC |
| NICHE | Neoadjuvant immunotherapy study of ipilimumab plus nivolumab in early-stage colon cancer |
| REGOMUNE | Regorafenib plus avelumab in MSS metastatic CRC |
| REGONIVO | Regorafenib plus nivolumab in advanced gastric or colorectal cancer |
